# Proteins analysed as virtual knots

**DOI:** 10.1038/srep42300

**Published:** 2017-02-13

**Authors:** Keith Alexander, Alexander J. Taylor, Mark R. Dennis

**Affiliations:** 1 H H Wills Physics Laboratory, University of Bristol, Bristol BS8 1TL, UK

## Abstract

Long, flexible physical filaments are naturally tangled and knotted, from macroscopic string down to long-chain molecules. The existence of knotting in a filament naturally affects its configuration and properties, and may be very stable or disappear rapidly under manipulation and interaction. Knotting has been previously identified in protein backbone chains, for which these mechanical constraints are of fundamental importance to their molecular functionality, despite their being open curves in which the knots are not mathematically well defined; knotting can only be identified by closing the termini of the chain somehow. We introduce a new method for resolving knotting in open curves using virtual knots, which are a wider class of topological objects that do not require a classical closure and so naturally capture the topological ambiguity inherent in open curves. We describe the results of analysing proteins in the Protein Data Bank by this new scheme, recovering and extending previous knotting results, and identifying topological interest in some new cases. The statistics of virtual knots in protein chains are compared with those of open random walks and Hamiltonian subchains on cubic lattices, identifying a regime of open curves in which the virtual knotting description is likely to be important.

Proteins are large, complex biomolecules exhibiting folded conformations whose precise form and stability are fundamental to their biological role^1^. As protein chains can be thought of as long, tangled curves, it is natural to ask if they can be *knotted*^2^,^3^,^4^,^5^,^6^,^7^. Mathematical knot theory only defines knots in closed, circular loops^8^, whereas the curves described by protein chain backbones have distinct endpoints; as *open chains* of carbon and nitrogen atoms, their knots may be ‘untied’ by smooth deformation. A degree of mathematical compromise is therefore required to determine whether a given protein chain may be considered knotted^4^,^9^; its termini must somehow be joined to make a closed curve, without distorting the protein’s configuration. Various closure constructions have been proposed^9^, generally giving similar results, and applied to protein chain catalogues^5^,^10^. These investigations have shown that knotting in proteins is in fact very rare^5^,^11^, likely owing to the chemical and mechanical difficulty of forming such structures making them evolutionarily disadvantageous^12^. Within a given protein curve, the knot structure may be deep (like a knotted shoelace) or shallow (unstable to perturbation), a key property that is related to the stability and importance of the knot.

Figure 1(a) shows a representation of a protein chain including alpha helices and beta pleated sheets. The protein backbone is approximated as a piecewise linear curve, not explicitly considering secondary structures, where each vertex representing a carbon alpha atom is either connected to its two neighbours or one neighbour at the termini, as shown in Fig. 1(b). The most obvious way of closing the backbone into a loop is to join its endpoints with a straight line, but such a crude procedure usually fails to give a knot representative of the protein^4^,^9^. A standard closure method^4^,^5^,^11^, which we refer to as *sphere closure*, is illustrated in Fig. 1(c): straight lines are continued from each backbone terminus to the same point on a sphere surrounding the curve. Each point on the closure sphere gives a closed curve of a specific knot type, which may be an unknotted circle. Nongeneric closures where the straight lines intersect the backbone are ignored. The sphere is given a large enough radius to avoid small-scale geometrical effects; in practice, the closing lines can be taken as parallel, closing ‘at infinity’. The closure sphere is partitioned into ‘islands’ of the different knot types resulting from closing at each point, and the knot type covering the greatest area is identified as the ‘knot type’ of the protein. The results of the ongoing *KnotProt* protein survey^5^ (as of Sep 2016) reveal that according to these definitions, 946 of the 159,518 sequence unique protein chains in the Protein Data Bank^10^ (PDB) are statistically knotted.

Here we present an alternative analysis of protein knots. Rather than *closing* the backbone curve in 3D, we consider *projections* of the open curve in every direction. Each projection is a 2-dimensional open *knot diagram*, a network of arcs intersecting at *crossing* points^8^. Three perpendicular projections of a simple open curve are depicted in Fig. 1(d). The endpoints of the diagrams in the red and green projections could be unambiguously joined and therefore be identified with usual closed knots. However, the endpoints in the blue projection are separated by a strand and cannot obviously be joined. Projections like this correspond to *virtual knots*, which generalize the ‘classical’ knots, capturing the open nature of the diagram via virtual knot types^13^. This identification of open diagrams with classical and virtual knots is called *virtual closure*.

The topological character of the open protein backbone chain is fully characterised by the distribution of different classical and virtual knots resulting from virtual closure over different projection directions. An advantage of this new method is that it allows a more subtle refinement of the knot distribution associated with an open curve, as the inclusion of virtual knots can better capture the conformations of backbones where tangling is evident but no single knot type dominates. This analysis appears particularly suitable for protein curves, and relates to the distinction between deep and shallow knotting. We quantify these changes, and suggest how these techniques could apply to specific other systems of open curves.

## Methodology and Results

### Projected open curves and virtual knots

**Figure 2:**
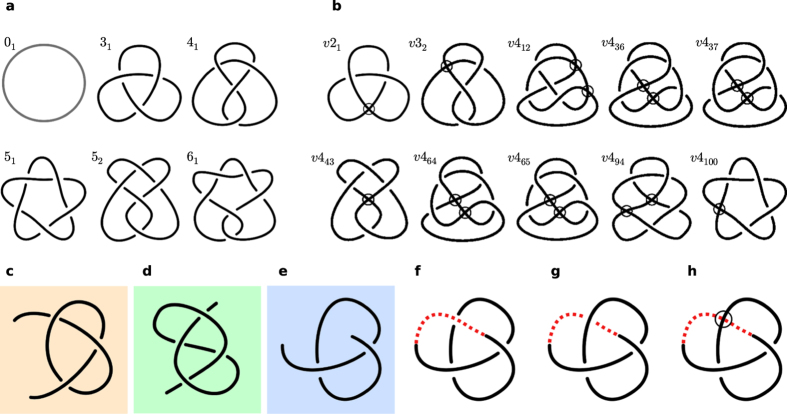
Classical and virtual knot diagrams. (**a**) The first six classical knots in the standard tabulation (including the unknot 0_1_); all but 5_1_ have been identified as dominant knot types in at least one protein under sphere closure^5^. (**b**) The virtual knots with *n* = 2,3,4 as tabulated in ref. 20, all of which can arise as virtual closures of open knot diagrams (i.e. the minimally genus one virtual knots, described in Supplementary Note 1). Virtual crossings are shown as circles. (**c**–**h**) show examples of open diagrams, which may be identified under virtual closure as classical or virtual knots. (**c**–**e**) are equivalent to the projections from Fig. 1(d). (**f**) and (**g**) show (**e**) closed with a classical arc passing above or below the intervening strands, forming an unknot 0_1_ and trefoil knot 3_1_ respectively, while (**h**) shows (**e**) closed instead with a virtual crossing to produce the knot *v*2_1_.

We now summarise some basic mathematics of knot and virtual knot classification^8^,^13^. A more complete summary of both classical and virtual knot theory is given in Supplementary Note 1. Knots are labelled and ordered in *knot tables*^14^,^15^,^16^,^17^ according to their *minimal crossing number n*, which is the minimum number of crossings a 2-dimensional diagram of the knot may have^8^. The closed knots with *n* crossings are labelled *n*_*m*_, where *m* is an effectively arbitrary index, not distinguishing enantiomeric pairs with opposite chirality (our analysis does not distinguish between such pairs, although it would be possible to do so). Some simple knots are shown in Fig. 2(a) such as the unknot 0_1_ (counted for completeness) and the trefoil knot 3_1_ (the only knot with *n* = 3). Composite knots, in which more than one knot is tied in a single curve, do not appear in protein chains^5^. A given knot has many possible conformations, which may have arbitrarily many crossings in projection. Equivalent conformations, which can be deformed into one another without cutting and joining, are called *ambient isotopic*; their diagrams can be related algorithmically by a sequence of *Reidemeister moves*, a set of local arc and crossing changes representing smooth deformation of a 3D curve^8^ (see Supplementary Fig. 1).

The knot type of a diagram is entirely determined by its sequence of crossings between arcs, which encodes its topological information. Open curve diagrams are technically not knots as they do not represent a closed loop (the endpoints cannot necessarily be joined without introducing extra crossings), but their mathematical structure is preserved by standard Reidemeister moves. Virtual knots were introduced by Kauffman^13^ to make mathematical sense of such incomplete lists of crossings (represented, for instance, by a Gauss code, discussed in Supplementary Note 1). As such, virtual knots are more abstract and general than open curve diagrams, but do correctly encode their topology; we describe other interpretations below.

Analysing an open diagram as a virtual knot is equivalent to closing its endpoints with an arc that makes *virtual crossings* with the other arcs; these do not distinguish over or under crossing. Since all the topological information is contained within the classical crossings, such a virtual closure represents ‘not closing’ the curve. Virtual crossings can be algorithmically transformed without changing the virtual knot type via an extended set of *virtual* Reidemeister moves (see Supplementary Fig. 1). A given open knot diagram has the same virtual knot type under all possible virtual closures, although this may still represent a classical knot. This procedure is illustrated in Fig. 2(c–e): in (c) and (d) the endpoints can be closed with no additional virtual crossings, in both cases representing the classical trefoil knot 3_1_, while in (e) there is no way to avoid crossing an intervening strand. Figure 2(f) and (g) show the ambiguity of classical closure, resulting in the unknot 0_1_ and trefoil knot 3_1_ respectively, while in (h) the virtual closure produces a single virtual knot. Open knot diagrams could instead be considered as *classical knotoids*^18^, whose isotopies are determined by augmented Reidemeister moves which forbid endpoints from passing over/under any strand of the curve; although knotoids form topological classes^18^,^19^ they have not yet been robustly tabulated (see Supplementary Note 1). Our virtual knots are equivalently virtual closures of the classical knotoids^19^.

Virtual knots are tabulated^13^,^20^ with the same ordering logic, but written here with a prefix ‘*v*’, i.e. *vn*_*m*_ where *n* is again the minimum classical crossing number. There is no relationship between the classical *n*_*m*_ and virtual *vn*_*m*_. As with the classical tabulation, all mirror-symmetric partners are considered equivalent. Not all virtual knots can arise from virtual closure of open diagrams, only those which have a diagram with all the virtual crossings adjacent, with no classical crossings in between (i.e. along the closure arc). The examples with up to 4 classical crossings are shown in Fig. 2(b). There are still many more of these than classical knots for given *n*: the classical (virtual) count is 1 (0) for *n* = 0; 0 (1) for *n* = 2; 1 (1) for *n* = 3; 1 (8) for *n* = 4, etc.

In practice, the knot type of a closed diagram is found through calculation of *knot invariants*^8^,^13^,^14^,^20^, which are functions of the diagram’s classical or virtual knot type. Most readily-calculated invariants fail to distinguish certain distinct knots^8^, so we identify types by the characteristic signatures of a set of invariants, calculated sequentially until the knot type is clear. It is more computationally efficient to calculate polynomial invariants at specific values rather than symbolically, and we consider them at certain roots of unity^21^. For classical knots, our invariants are: the *Alexander polynomial*^8^ Δ(*t*) at *t* = −1, *e*^2*πi*/3^, −*i*. For virtual knots we use the *generalised Alexander polynomial*^20^,^22^ Δ_*g*_(*s, t*) at (*s, t*) = (−1, *e*^2*πi*/3^), (−1, *i*), (*e*^2*πi*/3^, *i*); and the *Jones polynomial V(q*)^8^,^14^,^23^,^24^ at *q* = −1. Classical knots have Δ_*g*_ = 0.

**Figure 3:**
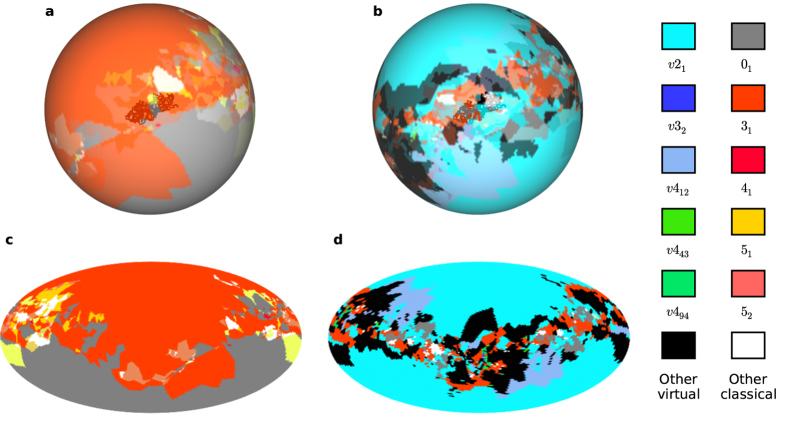
Classical and virtual knot types found amongst different projection/closure directions for a protein backbone chain. The protein backbone shown has PDB ID: 4K0B, chain A (*Sulfolobus solfataricus* S-adenosylmethionine synthetase)^49^. Each point is coloured according to the knot type (classical or virtual) found by closure/projection in that direction. Classical and virtual knot types are coloured according to the legend. (**a**) Classical knots resulting from 3-dimensional sphere closure in each direction. (**b**) Virtual knot types resulting from virtual closure of the diagram obtained from projection in each direction. (**c**) and (**d**) are Mollweide projections of (**a**) and (**b**). These images are constructed from sampling 10,000 directions in each case. Antipodal points on the sphere are always associated with the same knot type under virtual closure (up to possibly distinct mirrors for certain virtual knot types), but may produce different classical knots on sphere closure. This protein is considered strongly trefoil (3_1_) knotted under sphere closure, and strongly *v*2_1_ virtually knotted under virtual closure; it is an unusually strong exemplar of this behaviour, described in the following Section.

We analyse open curves in terms of the fractions of directions giving different knot types under sphere or virtual closure. Figure 3(a–d) demonstrates this for an example protein chain, for both closure methods: directions are coloured according to the knot types both on a sphere and in (area-preserving) Mollweide projection. In the sphere closure maps (b), (c), 59% of directions give a trefoil knot 3_1_, which therefore dominates and so this backbone was determined by ref. 5 to be 3_1_ knotted (alongside 34% unknots and 7% more complex knots shown by the smaller islands). Much of the area identified as 0_1_ or 3_1_ under sphere closure in (c), becomes, in the corresponding virtual closure map (d), the virtual knot *v*2_1_ in 54% of different projections. This curve therefore has strong virtual character, and its virtual knot type reflects the ambiguity of the open curve between the unknot and trefoil knot.

### Analysis of the Protein Data Bank

We now present the results of our survey of knotting in the Protein Data Bank (PDB)^10^, using both sphere closure and virtual closure. We analyse the same set of protein chains indexed by the KnotProt database^5^ (i.e. taking only each sequence unique chain in a given protein and rejecting some chains with breaks in their recorded structures, see Methods), additionally discarding chains obsoleted in the PDB by more recent measurements. This gives a total of 159,518 distinct protein chains for analysis, from the 121,532 full PDB structures. The chain records can still contain breaks where their structure is uncertain, which we close with straight lines. For each chain, we consider 100 different closure/projection directions (approximately uniformly distributed on the sphere following the method of ref. 25), considered sufficient for reasonable numerical confidence at acceptable computational cost^4^.

The sphere closure analysis of KnotProt found 946 knotted chains, including 871 occurrences of 3_1_, 45 of 4_1_, 27 of 5_2_ and 3 of 6_1_ (at time of comparison: Sep 16). Our corresponding analysis gives instead 972 knotted chains, including 894 of 3_1_, 48 of 4_1_, 27 of 5_2_ and 3 of 6_1_, including all but one of the KnotProt-identified chains, and 27 additional knot detections. These discrepancies appear to arise from small differences in methodology, particularly in rare occasions where very severe chain breaks are present; 17 of our extra detections are considered knotted by one or both of the alternative protein knots databases pKNOT^26^, or Protein Knots^27^. We therefore consider that our sphere closure methodology accurately detects protein knotting for the purpose of comparison with virtual closure.

In the sphere closure results, each open chain is associated with the knot type most commonly occurring in different directions (i.e. the modal average). Although this methodology is natural, this can miss certain interesting cases; for instance, a chain closing to the unknot in 40% of directions, 3_1_ in 30% and 4_1_ in 30% would be considered unknotted, despite being some knot in the majority of closure directions. Such cases are much more frequent under virtual closure, since many more knot types are possible and the resulting maps are correspondingly more complex, as shown in Fig. 3. We therefore introduce new classes of knotting associated with open chains, defining an open chain to be *unknotted* only if it appears to be 0_1_ in over 50% of closure directions; otherwise it is knotted, in some sense. For sphere closure, if a single (nontrivial) knot type occurs in at least 50% of directions we call this *strongly knotted*, while if the sum of different nontrivial knot types occurs for at least 50% of directions, but no single type does, we call this *weakly knotted*. 968 of the 972 protein knots discussed above are strongly knotted according to this definition, and 7 further chains are weakly knotted. The choice of threshold at 50% knotted is somewhat arbitrary, and the number of curves identified as unknotted rises (falls) as it is increased (decreased).

**Figure 4:**
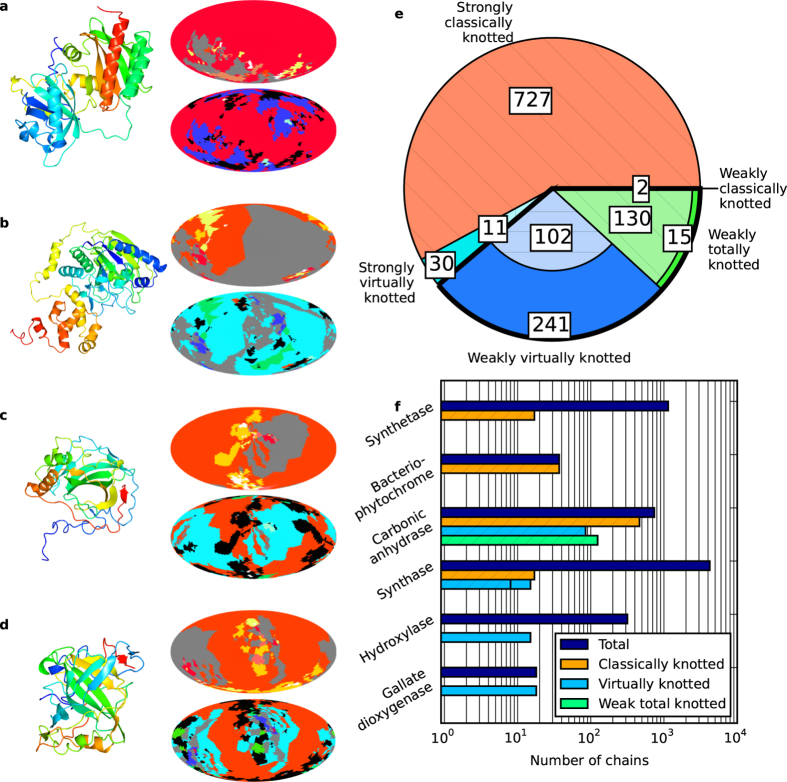
Results of virtual closure analysis for knotting in the Protein Data Bank. Knotting classifications follow the main text; strong classical (virtual) knotting where more than 50% of projections form the same classical (virtual) knot type; weak classical (virtual) knotting when over 50% of projections form classical (virtual) knots but no single knot type dominates, and weak total knotting where the unknotting fraction does not exceed 50% but no other specific class dominates. (**a**–**d**) Examples of knot type maps (see Fig. 2) for protein chains in these different classes, coloured according to the legend of Fig. 3. The upper (lower) map in each case shows the results of sphere closure (virtual closure): in (**a**) PDB ID: 4E04, chain A (*Rhodopseudomonas palustris* RpBphP2 chromophore-binding domain)^50^, which is classically knotted in both cases; in (**b**) PDB ID: 3WKU, chain B (*sphinogobium sp. SYK-6* extradiol dioxygenase)^51^, which is not knotted under sphere closure but is strongly virtually knotted under virtual closure; in (**c**) PDB ID: 4XIX, chain A (*Chlamydomonas reinhardtii* carbonic anhydrase)^52^, which is knotted under both sphere and virtual closure, weakly virtually knotted in the latter; and in (**d**) PDB ID: 3KIG, chain A (*Homo sapiens* carbonic anhydrase II mutant)^53^, which is knotted under sphere closure and exhibits weak total knotting on virtual closure. (**e**) Numbers of protein chains in each knotting class under virtual closure. (**f**) Knot types found amongst selected categories of protein chain names, and their distribution amongst knotting classes. In (**e**) and (**f**), hatched areas represent chains which were also identified as knotted under sphere closure.

Under virtual closure, different projections of an open curve can give a mixture of virtual and classical knot types. We refine the distinction of strong and weak knotting to distinguish classical and virtual knotting. A chain is *strongly classically (virtually) knotted* when a single classical (virtual) knot type appears in more than 50% of projection directions. A chain is *weakly classically (virtually) knotted* if no knot type is so individually common, but the sum of directions closing to classical (virtual) types contributes to over 50% of projection directions. A chain where the sum of classical and virtual types adds to over 50%, but neither does separately, is *weakly totally knotted*. The weak classes represent curves whose projections have significant topological character not represented by a single knot type. Examples of protein chains according to these classifications are shown in Fig. 4(a–d), and the identifications may vary significantly from the results obtained by sphere closure: (a) is strongly classically knotted according to both analyses; (b) was unknotted on sphere closure but is strongly virtually (*v*2_1_) knotted on virtual closure; (c) was strongly 3_1_ knotted on sphere closure but is weakly virtually knotted on virtual closure; and (d) was strongly 3_1_ knotted on sphere closure but on virtual closure is weakly totally knotted.

Under virtual closure we find 1258 protein chains knotted according to our definition, 283 more than under sphere closure. The proportions of different classes are summarised in Fig. 4(e). Most of these protein chains are again strongly classically knotted (727 cases, all of which were also strongly classically knotted under sphere closure, and mostly the knot 3_1_), and weak classical knotting is still negligible (2 cases, compared to 7 under sphere closure). Strong virtual knotting is much less common than strong classical knotting, occurring in 41 cases, from which 30 are unknotted under sphere closure. These are cases where, under sphere closure, two classical knot types compete with comparable areas (in all but one case the competition is between 0_1_ and 3_1_); the virtual knots are therefore strongly *v*2_1_ knotted (the other is *v*4_43_ between classical types 0_1_ and 5_2_).

The remaining protein chains are weakly knotted in some form; 343 are weakly virtually knotted (around a third of which were unknotted under sphere closure), and 145 are weakly totally knotted (most of which were dominated by a classical knot under sphere closure). This is demonstrated in the curve of Fig. 4(c), whose sphere closure map suggests little of the complexity evident in its virtual closure map; this feature is typical of the weak virtual knots, which often appear unknotted under sphere closure. These knots may be interpreted as being rather shallow, as small modifications to the chain might significantly affect the maps. The weakly totally knotted chains are similar but with the classical knots a little deeper in the chain, as in the example of Fig. 4(d), where the clarity of the chain’s trefoil knot character is muted but not removed under virtual closure.

Our designations of strong and weak knotting crudely capture the forms of knotting and tangling exhibited in protein backbone curves, with physical implications for the depth of the knots in the chain. The distribution of these classes is uneven amongst the protein chains; for instance, all 46 examples of 4_1_ under sphere closure remain strongly 4_1_ under virtual closure, suggesting consistently small virtual character. Knotting is also not equidistributed amongst different protein classes: Fig. 4(f) shows a breakdown of the the different classes of knotted open chain by protein chain name, for families in which knotting has previously been observed to cluster^5^, as well as families where new virtual character appears. Virtual knotting appears but is not dominant amongst carbonic anhydrases, in which the knots are known to be rather shallow, and all knots found under virtual closure also appear under sphere closure. In contrast, the virtual knots amongst synthases are almost all newly identified, with previously discovered strong classical knots being deep enough to remain unchanged by the analysis. Further, the families of hydroxylases and gallate dioygenases contain several examples of virtual knotting, and neither family showed any evidence of knotting under sphere closure, although both of these families represent small groups of geometrically similar proteins. It is unsurprising that the levels of topological complexity are reasonably consistent among members of the same protein families, as they arise from consistent features in their secondary and tertiary structures, but it is important that virtual knotting has its own distribution among protein chain names, distinct from that of classical knotting.

### Comparison with random open chain ensembles

The virtual closure technique may be applied to describe the knotting of any open space curve. In order to understand better whether the proportion of virtually knotted proteins is typical amongst families of open curves, and to investigate what this means geometrically, we perform a preliminary virtual knotting analysis for two other families of random open curves: open random walks, and open subchains of Hamiltonian walks on a cubic lattice. We use a simplification of the scheme in the previous section, considering an open curve as ‘knotted’ if over 50% of directions yield a knot on sphere closure (i.e. strong or weak classical knotting), and ‘virtually knotted’ if over 50% of projection directions are virtually knotted (i.e. strong or weak virtual knotting). The main parameter against which knotting is compared is *closing distance fraction* (CDF)—the distance between the curve’s endpoints divided by its total length—which varies from 0 for a closed loop, to 1 for a straight line.

Random walks consist of a sequence of random linear steps, whose limiting, long-length statistical behaviour is that of Brownian motion. For sufficiently long walks, the statistics are independent of the specific model, tending towards the characteristic Brownian fractal behaviour^28^. The probability of knotting in closed random walks has been well investigated^29^. Random walks do not model proteins well, but nevertheless are good models for other physical systems^21^,^29^,^30^, and are a convenient comparison model for open chains in the absence of physical constraints.

**Figure 5:**
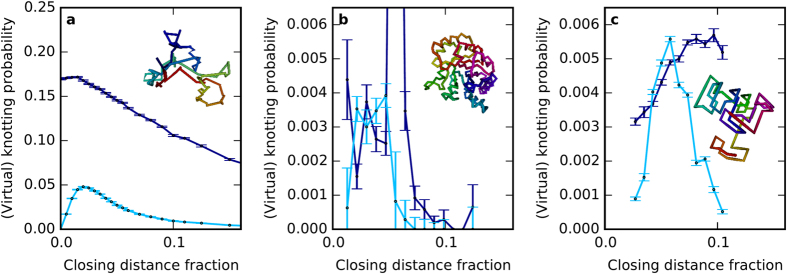
Knotting and virtual knotting probabilities in different open curve ensembles. The closing distance fraction (CDF) is the ratio of the distance between the open curve’s endpoints with respect to the total curve length. The lines compare the primary properties of closure and virtual knotting: the dark blue line shows knotting probability under sphere closure (considering an open curve as ‘knotted’ if over 50% of directional closures yield a knot); while the light blue line shows virtual knotting probability (considering an open curve as ‘virtually knotted’ if over 50% of directional closures yield a virtual knot, counting both strong and weak virtual knotting). Knotting probabilities are plotted for (**a**) 6 × 10^6^ open random walks of length 100; (**b**) all 159,518 proteins analysed in the previous Section, with various lengths and binned according to CDF; (**c**) 5.5 × 10^6^ length-75 subchains of Hamiltonian walks on cubic lattices of side length 6, binned by CDF. In (**b**), the sharp peak at a CDF of 0.047 reaches a height of ~0.033, but contains no subtler structure and so the plot is not scaled to show its shape, discussed in the main text. In (**c**), the fluctuations reflect correlations implicit in the lattice. In each figure, the inset shows a typical example of the curve ensemble, coloured red to blue by hue along its length to distinguish different regions of the curve. Error bars represent the standard error on the mean probability of the knot statistic.

Figure 5(a) shows the statistics of knotting upon sphere and virtual closure for a set of random walks with 100 steps generated via the method of ref. 31, with inset showing a sample random walk. The advantage of this particular ensemble is that the CDF can be directly controlled. For all distances knotting is significantly more common than virtual knotting; both are most probable around a CDF of 0.025, where about 5% of the random walks are virtually knotted, but even at this value classical knotting is at least 3.5 times as common. Random walks of different lengths (not shown) share similar behaviour. These results are not surprising as knots in random walks can easily be small, localised deep within the chain.

This contrasts strongly with the behaviour for proteins, shown in Fig. 5(b), where all knotted protein chains from the previous Section are combined despite their backbones being of many different lengths (from tens to thousands of angstroms, and up to ~3300 carbon atoms in the backbone chain). The comparatively small number of protein chains mean the statistics are only useful for qualitative comparison. Nevertheless, virtual knotting appears far more likely relative to classical knotting across all closure fractions, possibly becoming more dominant around a CDF of 0.025. The exception is a large peak in knotting probability around a CDF of 0.047; this represents primarily carbonic anhydrases, many of whose lengths cluster around this value and which are observed in the literature to have an uncommonly high knotting probability^5^,^32^, but these appear to be an unusual exception to the virtual knotting trend.

Unlike random walks, protein backbones are characterised by relatively compact geometries (e.g. the inset to Fig. 5(b)), and aspects of this can be reproduced by simple mathematical models of random chains. In Fig. 5(c), we give the results for one such model: a subchain of a Hamiltonian walk^11^, that is, a path on a cubic lattice of fixed size, visiting every vertex once and every edge no more than once. Such curves form a confined, folded structure due to the strict boundaries of the finite lattice. The geometry and topology of proteins are best approximated by a much shorter subchain of the walk, reducing the effect of the lattice confinement. Random lattice walks of this type can be efficiently generated up to lattice side lengths of at least 10 ref. 33.

Figure 5(c) shows the knotting and virtual knotting sampled from 5.5 × 10^6^ random Hamiltonian subchains with length 75 on a cubic lattice of side length 6 (total Hamiltonian path length 255), with these parameters chosen to approximate the knotting probabilities in Fig. 5(b). For reference, the radius of gyration of subchains with this length corresponds to CDF ~ 0.036. Virtual knotting here is strong relative to classical knotting, comparable to proteins but very unlike random walks; the probability of virtual knotting exceeds that of classical knotting across the small range 0.04 ≲ CDF ≲ 0.055. This trend appears to be highly robust to different parameters; even for complete Hamiltonian chains, in which knots are very common, virtual knotting exceeds classical knotting over approximately the same range. These results emphasise that virtual knotting is a generic feature of certain geometrical classes of curves, arising from relatively weak geometric constraints even in the absence of the physical complexity of protein chains.

## Discussion

We have shown that the backbones of protein chains, as well as other open curves, can be described topologically in terms of virtual knotting. Through the method of virtual closure, projections of open chains are found to have a much wider set of topological classes than the classical knots in closed curves, and proteins provide examples of many different virtual knot types. Nevertheless, virtual knotting dominates relatively few proteins, and the virtual knot types which do occur are only the simplest of the possible virtual knots. In some cases this can be thought of as representing a more nuanced characterisation of ‘almost’ knotted curves, softening the binary distinction between knotting and unknotting imposed by traditional closure methods. In the analysis of proteins the most dominant virtual class is the weak virtual knots, where no single type is dominant, but fewer than 50% of projected diagram directions are unknotted. These curves are the most topologically ambiguous, and cannot be associated with a definite knot type. Curves are otherwise strongly knotted when a single classification dominates, or described by other classes of weak knotting for different combinations of virtual and classical knot contributions.

Although these broad classes capture some distinction in the way open curves tangle, they do not quantify the rich structure of knot types in the projected map, whose other properties may be key to understanding the 3D spatial conformation of the open chain. Including virtual knots may be a step towards this because, in the spherical maps, they generally occur in between classical knot types (seen clearly in Figs 3 and 4(b–d)), even in chains which are mostly unknotted. An example system in which this extra structure may be important is the dynamics of (un)knotting in an open curve over time; one might study how islands of virtual knotting behave in the time sequence of spherical maps as a deep knot (un)ties in an open curve.

We have seen that protein chains express several geometrical properties that might be expected to encourage virtual knotting: as they fold, they curve and twist into relatively small, chemically bound structures such that their projections have many crossings; the endpoints of the protein backbone are often within or near the surface of the structure, such that projections in different directions produce distinctly different knot diagrams; and the physical limits on their curvature and overall tangling mean that knots are rarely unambiguous local structures but inherently involve the entire protein chain. This is not true for random walks, and indeed we found virtual knotting to be less significant in them. Hamiltonian subchains do share some of these properties, and were found to be particularly strongly virtually knotted. We expect that virtual knotting analysis will therefore be relevant in other physical systems of open curves with compact configurations. A mechanism that might encourage virtual knots in physical systems is tight confinement, such as that of a curve confined within a sphere (e.g. DNA within a viral capsid^34^,^35^), or between adjacent planes^36^,^37^.

Although our discussion has focused on the immediate statistics of virtual knotting in protein backbone chains, of course the analysis only requires that the curves are open-ended. Virtual closure refines rather than replaces existing methods of analysing knotting in open curves, and can be applied more widely in place of sphere closure. One example is slipknotting, where curves contain knotted subchains that are ‘unthreaded’ by the the rest of the curve, many examples of which have been found in proteins^5^,^38^. Virtual knots would again be anticipated to occur at transitions between different classical knot types in a slipknotting fingerprint analysis. The virtual closure methodology could be extended to multiple open curves, which would virtually close to virtual links, and may even extend to other knot- and link-like objects^32^,^39^,^40^,^41^ such as protein lassos^42^,^43^,^44^.

## Methods

### Knot detection by sphere closure of open curves

For each open chain (here, a protein backbone or random walk), each direction (point on a sphere around the curve) is associated with a type of knot. For the sphere closure analysis, the endpoints of the open curve are closed by extending them ‘to infinity’ in this direction, giving a closed curve of a specific classical knot type. In practice, the 3D chain is projected in the plane perpendicular to this direction, then the diagram closed with a straight line that passes *over* every intervening arc of the diagram. Each open curve is projected and analysed in 100 approximately uniformly distributed closure directions, chosen using the algorithm of ref. 25. Previous work has verified that 100 closure directions is usually sufficient to determine the significant statistical behaviour of closures in different directions^4^, and so alternative approximately-uniform samplings should reproduce the same statistics. For each projection, the resulting knot diagram is algorithmically simplified using Reidemeister moves (see Supplementary Note 1), then the knot type identified through the calculation of knot invariants as described in the main text. The invariant used is the modulus of the Alexander polynomial, |Δ(*t*)|, evaluated at each of *t* = −1, *t* = *e*^2*πi*/3^ and *t* = *i*, computed using a standard scheme^29^. The Alexander polynomial is used because it can be calculated in polynomial time in the number of crossings of a knot diagram (more discriminatory invariants are harder to calculate), but it is still sufficient to distinguish unambiguously knots with up to at least 8 crossings; more complex knots may have invariants taking the same values, but these complex conformations are rare and never dominate in protein chains (for instance, the next knot with the same Alexander polynomial as the trefoil knot 3_1_ has 13 crossings, and no simpler knot agrees at the roots of unity we consider). For simple knots this choice of three evaluation values is just as discriminatory as the full Alexander polynomial, but more convenient for numerical calculation.

### Knot detection by virtual closure of open curves

For the virtual closure analysis of open curves, we select the same 100 projection directions as above (these appear to be sufficient to distinguish classical and virtual knot types as in the sphere closure analysis). The projected diagram in a given direction is virtually closed and again simplified algorithmically using both classical and virtual Reidemeister moves (see Supplementary Note 1). Virtual knots require different invariants, we use the generalised Alexander polynomial Δ_*g*_(*s, t*) at certain pairs of arguments (*s* = −1, *t* = *e*^2*πi*/3^), (*s* = −1, *t* = *i*) and (*s* = *e*^2*πi*/3^, *t* = *i*). Unlike the classical knots, even the simple virtual knots *v*2_1_, *v*3_1_ and *v*4_94_ have equal Δ_*g*_(*s, t*) = (−*s*^−2^ + *s*^−1^)*t*^2^ + (*s*^−2^ − 1)*t*^−1^ + (−*s*^−1^ + 1). In these cases we additionally calculate the Jones polynomial *V(q*) at *q* = −1 ref. 8, which requires exponential time in the crossing number but unambiguously distinguishes all these examples. Some more complex virtual knots would also be ambiguous to these measurements but, as with classical knots in sphere closure, are far more complex than those appearing in protein chain closures. Some virtually closed diagrams represent classical knots, in which case Δ_*g*_(*s, t*) = 0 and the Alexander polynomial is used as above. These cases are still occasionally complex virtual knots with vanishing Δ_*g*_, so we further calculate whether the classical knots produced from over- and under-closure of the virtual crossing arc are the same; although not proven, we anticipate that if their knot types differ the diagram likely represents a virtual knot, whose type we do not identify. In practice, such cases make up a negligible fraction of total projections and do not limit the analysis.

### Numerical analysis of protein backbone chains

The set of protein chains analysed are taken from the knotted and unknotted lists given under the database statistics section of the KnotProt web server^5^. These take one sequence unique chain from homomultimeric complexes and reject some chains that are detected as knotted only due to severe breaks in the recorded backbone, as determined by KnotProt. We only analyse the chains in this set that have not been made obselete by newer measurements. The protein chains are obtained from the list of all resolved protein molecules in the Worldwide Protein Data Bank (PDB)^45^. In each case the.pdb protein record is downloaded and parsed using ProDy^46^. In particular, we parse the atomic coordinates of each carbon alpha atom, and reconstruct the protein backbone by connecting these sequentially with straight lines as an approximation of the true NCCNCC backbone. In some cases there are still chain breaks where residues are missing in the PDB record, and here the distant carbon alphas across any breaks are connected with straight lines to create one, continuous open curve. Although this does not reproduce the exact protein geometry, most chain break distances are well below ~20Å (~5 carbon alpha separation distances) and do not significantly affect the recovered structure. 5475 of the remaining chains have large break distances above 20Å (although significantly larger breaks are very unusual and not statistically significant), of which 88 appear as some type of knot in our analysis. We also ignore heteroatom structures. Where protein chain names are referenced in the text, these are as recorded in the PDB. Protein ribbon structure images were created using CCP4mg^47^.

## Additional Information

**How to cite this article:** Alexander, K. *et al*. Proteins analysed as virtual knots. *Sci. Rep.* **7**, 42300; doi: 10.1038/srep42300 (2017).

**Publisher's note:** Springer Nature remains neutral with regard to jurisdictional claims in published maps and institutional affiliations.

## Supplementary Material

Supplementary Information

## Figures and Tables

**Figure 1:**
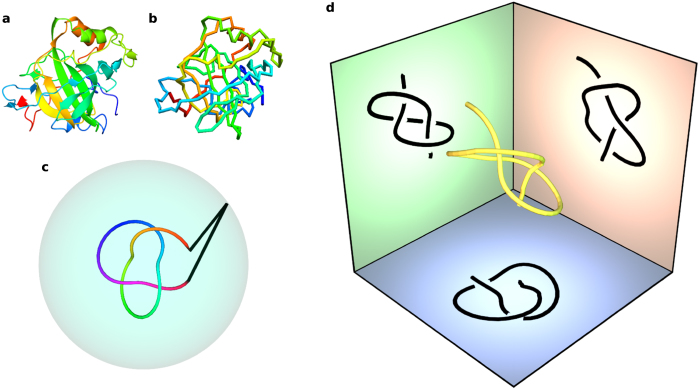
Protein backbone structures as open knotted space curves. (**a**) Backbone and some secondary structure of the protein with PDB ID 4COQ, chain A (*Thermovibrio ammonificans* alpha-carbonic anhydrase)^48^. (**b**) The backbone chain of carbon alpha atoms of the same protein as a piecewise-linear space curve. The colouring along the chain distinguishes different regions and does not have physical meaning. (**c**) The closure of an open curve from its termini to a point on a surrounding sphere by straight lines. (**d**) A 3-dimensional open curve and its planar projections in three perpendicular directions; each projection here gives an open knot diagram, where each crossing in the projection indicates which strand passes over or under the other. In this example, each projected knot diagram represents one of two different knot types, as explained in the text. Our analysis of open curves uses many such projections in different directions.
