# Supplementary material for: Proteins analysed as virtual knots
Source: Sci Rep. 2017 Feb 13;7:42300. doi: 10.1038/srep42300 (PMC5304221; doi:10.1038/srep42300)
Supplement: Supplementary Information [file srep42300-s1.pdf]

Supplementary Information: Proteins analysed as virtual knots

Keith Alexander, Alexander J Taylor, and Mark R Dennis

H H Wills Physics Laboratory, University of Bristol, BS8 1TL, UK

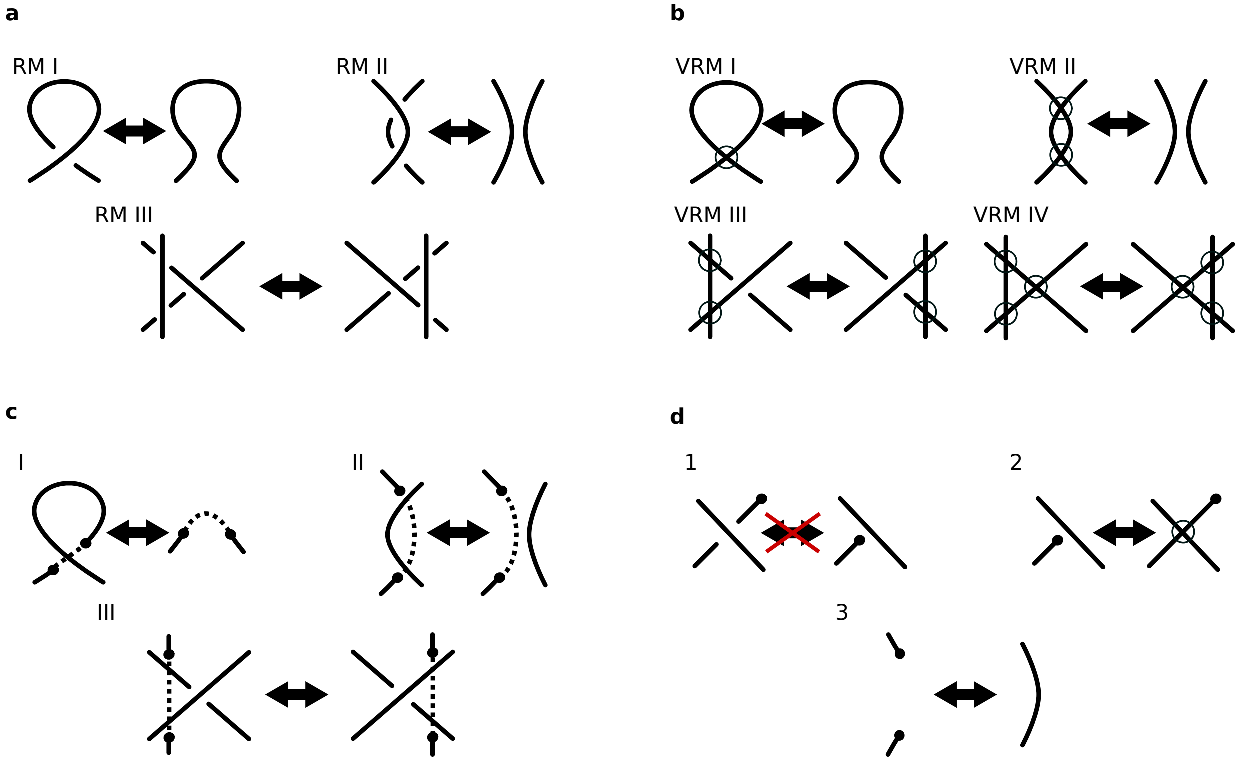

Supplementary Figure 1. Classical and virtual Reidemeister moves, and other algorithmic operations on open knot diagrams. The Reidemeister moves are local modifications to adjacent crossings of knot diagrams that do not change their topology. For each move, the rest of the curve (not shown) is assumed not to interact with the depicted region. A suitable combination of Reidemeister moves can (alongside planar isotopies) transform a given knot diagram to any other representing the same knot. (a) shows the classical Reidemeister moves involving only classical crossings, with standard labellings. (b) shows the virtual Reidemeister moves (virtual crossings are circled), which can involve changes in both classical and virtual crossings. (c) shows analogues of the virtual Reidemeister moves as closures of open curve diagrams with endpoints, in which case the Reidemeister changes clearly do not affect the topology resulting from virtual closure of the open strand. (d) highlights other relations that can be applied to open curve diagrams, whose application does not affect the virtual knot type resulting from virtual closure (disallowing moves 2 and 3 here reproduces the knot diagram relations of classical knotoids [1]). In (c) and (d), each endpoint of the open endpoints of the open curve is marked by a black circle.

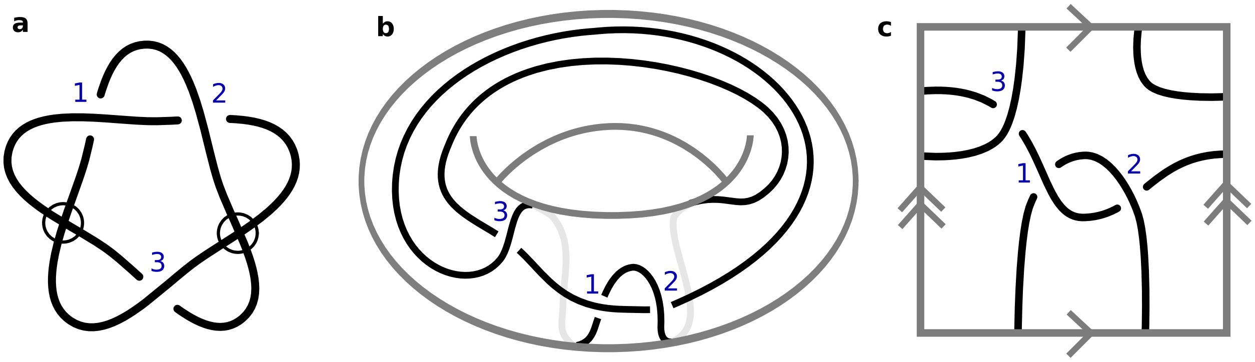

Supplementary Figure 2. Virtual knot $v3_7$, a virtual knot which cannot be formed from the closure of a projected open curve. The usual virtual knot diagram is shown in (a) while the presentation in (b) is depicted on the surface of a torus, on which the arcs of the diagram can be drawn with only the three classical crossings (light grey lines mark where the diagram passes through the hole of the torus, not normally visible). (c) shows a more standard diagrammatic representation on the flat torus, i.e. the square with opposite sides identified. For direct comparison, the crossings are numbered the same way in each diagram.

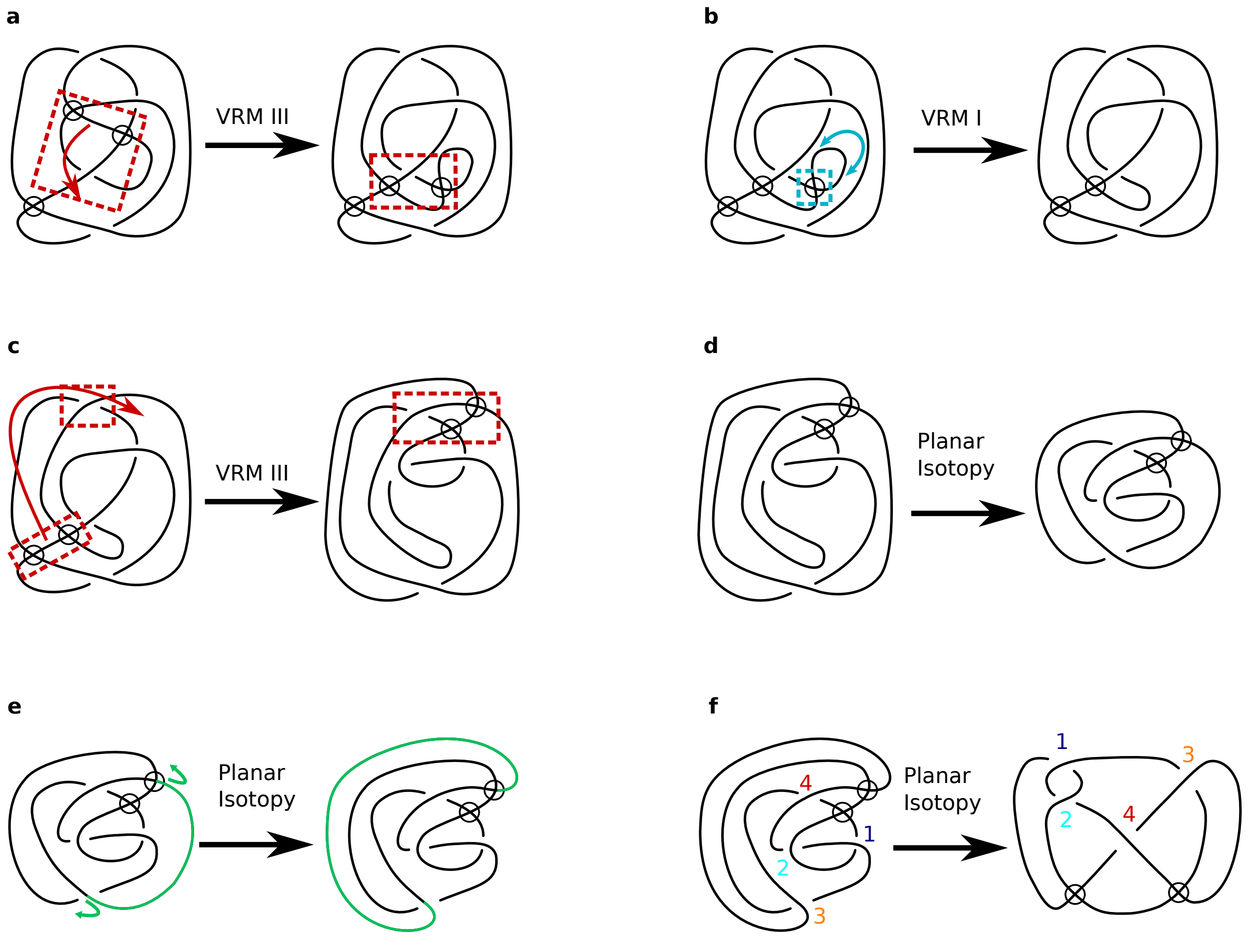

Supplementary Figure 3. Transformation between two depictions of the virtual knot $v4_{64}$. The initial conformation in (a) is that depicted as $v4_{64}$ in the virtual knot table of [2]. This conformation could not arise from virtual closure of an open curve, as its virtual crossings do not lie sequentially along a single arc. An alternative presentation of this knot from the genus one table of [3] (labelled there as 4_8), is shown in (f), and does have such a conformation, although it is difficult to see by eye that this is the same knot as $v4_{64}$. (b)-(e) show how (a) may be transformed to (f) by a combination of virtual Reidemeister moves and planar isotopies of the knot. In (e), the planar isotopy moving the green strand across the knot is not directly allowed by the virtual Reidemeister moves, but as the knot diagram is implicitly drawn on $\mathbb{S}^2$ this represents the strand passing 'behind' the sphere (or on the plane, passing through infinity). In general it is difficult to test whether two (virtual) knot diagrams can be related this way, hence the calculation of knot invariants which remove the need for diagrammatic manipulation.

Knot	$\Delta(-1)$	$\Delta(e^{2\pi i/3})$	$\Delta(i)$	$\Delta_g(-1, e^{2\pi i/3})$	$\Delta_g(-1, i)$	$\Delta_g(e^{2\pi i/3}, i)$	$V(-1)$	$\Delta_g(s,t)$
0_1	1	1	1	0	0	0	1	0
3_1	3	2	1	0	0	0	3	0
4_1	5	4	3	0	0	0	5	0
5_1	5	1	1	0	0	0	5	0
5_2	7	5	3	0	0	0	6	0
6_1	9	7	5	0	0	0	9	0
$v2_1$	-	-	-	3	4	5	2	$s^2 + s^2/t + st - s/t - t - 1$
$v3_2$	-	-	-	3	4	5	4	$s + s/t + t - 1/t - t/s - 1/s$
$v4_{12}$	-	-	-	3	8	5	5	$s^2/t + s^2/t^2 - st - s/t - 2s/t^2 - t^2 + t - 1/t +$ $1/t^2 + 2t^2/s + t/s + 1/st - t^2/s^2 - t/s^2$
$v4_{36}$	-	-	-	3	8	9	4	$t - 1 - 1/t + 1/t^2 + t^2/s - 2t/s + 2/st - 1/st^2 -$ $t^2/s^2 + t/s^2 + 1/s^2 - 1/s^2t$
$v4_{37}$	-	-	-	0	4	8	2	$s^4 - s^4/t^2 + s^3t + s^3/t^2 - s^2t + s^2/t - st^2 - s/t + t^2 - 1$
$v4_{43}$	-	-	-	7	8	9	5	$s^3 + s^3/t + s^2t - s^2/t - st - s$
$v4_{64}$	-	-	-	3	4	0	4	$s^2/t + s^2/t^2 - s/t - s/t^2 + t^2/s + t/s - t^2/s^2 - t/s^2$
$v4_{65}$	-	-	-	3	8	9	5	$-s^2t + s^2 + s^2/t - s^2/t^2 - st^2 + 2st -$ $2s/t + s/t^2 + t^2 - t - 1 + 1/t$
$v4_{94}$	-	-	-	3	4	5	5	$s^3 + s^3/t + s^2t - s^2/t - st - s$
$v4_{100}$	-	-	-	3	0	8	4	$s^4 + s^4/t + s^3t - s^3/t - s^2t + s^2/t + st - s/t - t - 1$

Supplementary Table 1. Table of numerical knot invariants for each knot shown in Fig. 2(a) and 2(b) of the main text. Included are $\Delta(t)$, the Alexander polynomial at the numerical values used, $\Delta_g(s,t)$ the generalised Alexander polynomial, both at the numerical values used and the full symbolic expression, and $V(q)$ the Jones polynomial. Virtual knots have no Alexander polynomial and so these columns are omitted. In the cases where chiral mirrors give different knots, only one mirror is given.

Supplementary Note 1: Topological Background

Classical Knot Theory

Here we summarise some extended details of mathematical knot theory as used in deriving the results of the main text. Further details can be found in standard elementary texts [4–6].

Classical knot theory deals with embeddings of the circle, S^1 (i.e. closed, non-intersecting curves), in three-dimensional space $\mathbb{R}^3$. Any given embedding has a distinct *knot type*, which is invariant under ambient isotopies (it may change only when the curve passes through itself). It is usual to represent knots using a two-dimensional planar *knot diagram*, which can be thought of as a plane projection of the three-dimensional space curve, annotated with the extra information of which strand passes over the other at each self-intersection of the diagram (called a *crossing*). All the information about the three-dimensional knot type is contained in such a diagram, and smooth deformations (i.e. ambient isotopies) of the three-dimensional space curve lead to smooth isotopies of the knot diagram, which may change the configuration of the crossings. In two-dimensional knot diagrams, the changes are represented by combinations of *Reidemeister moves* as in Supplementary Fig. 1(a); applying these local moves in conjunction with *planar isotopies* of the knot diagram can transform between any two diagrammatic representations of the same knot, and equivalently any ambient isotopy of a closed three-dimensional space curve corresponds to a combination of planar isotopies and Reidemeister moves in any projection of the knot.

The standard tabulations of knots, in *knot tables* as discussed in the main text, are ordered according to their *minimal crossing number* n – the smallest number of crossings a diagram of the knot can have. For instance, the trivial circle can be projected to a plane without self intersection (i.e. no crossings), and so has minimal crossing number $n = 0$ and is labelled 0_1. There are no knots with $n = 2$, and one with $n = 3$, the trefoil knot, denoted 3_1. The labelling n_m continues, where m is an arbitrary index amongst knots with the same n. These labels are standard, following original tabulations up to $n = 10$ published over 100 years ago, with more recent extensions using consistent indices [5, 7, 8]. Some simple knots from these tabulations are shown in Fig. 2(a). 0_1 (the *unknot*), then 3_1, 4_1, etc. The knots appearing in knot tables are *prime knots*; *composite knots*, made up of two or more prime knots tied in the same curve, are also possible and are tabulated according to the composition of their prime factors [4]. All the tools of knot theory apply equally to composite knots, but they do not occur significantly in any known protein chain, and are not considered further here.

It is natural to follow the curve of a knot, which endows an *orientation* to the knot (choosing an orientation is an arbitrary choice that does not affect the results of topological calculations). Observing the relative orientation of the strands at a crossing determines the *sign* of the crossing, either positive or negative. A crossing has the same sign even if the curve's orientation is reversed. The minimal diagram of a figure-8 knot 4_1 has two positively signed crossings and two negatively signed, and in fact is isotopic to its mirror image. On the other hand, all three crossings of the minimal trefoil knot 3_1 have the same sign, and are all reversed on its mirror image. Knots such as the trefoil are thus *chiral knots*, and this chirality not directly represented in the tabulation (i.e. there are two enantiomeric trefoil knots which cannot be be smoothly deformed into one another). Other chiral knots are 5_1, 5_2 and 6_1 in Fig. 2(a) of the main text; the others are achiral. We do not distinguish between chiral knot pairs in our analysis, although knot invariant quantities such as used to distinguish knots below could be used to do so.

In practice the knot type of a space curve is determined as follows. First the curve is projected to a 2D knot diagram, which contains all the topological information in its ordered set of signed crossings along the curve. Several topological notations representing this information are standard [4, 5]; we use below the *Gauss code*, constructed from an arbitrary starting point and orientation for the curve. As each new crossing is encountered along the curve, it is labelled $1, 2, \ldots$ in order as it is encountered. The Gauss code is the ordered list of these crossing numbers as they occur along the curve, together with whether the curve passes over or under the intersecting strand, represented by using a positive number in the former case and negative in the latter (this is not the same as the crossing sign); each crossing must be encountered exactly twice before reaching the original starting point, once positive and once negative. For instance, a Gauss code for a minimal diagram of the trefoil knot 3_1 is $1, -2, 3, -1, 2, -3$, and for a minimal figure-8 knot 4_1 is $1, -2, 3, -1, 4, -3, 2, -4$. It is obvious that changing the starting point on the curve cyclically permutes the crossings encountered, but all the Gauss codes obtained this way, or by changing numeric labels (as long as each crossing retains a unique label) represent the same knot diagram. The Gauss code written in this way also does not specify the chirality of the original three-dimensional curve, this information is contained in the local twisting of the two strands around one another and is sometimes included in extended Gauss code notations. Crossings which can be removed by Reidemeister moves I and II can be easily identified in a Gauss code; if crossing k occurs adjacent to itself, $\pm k, \mp k$ then it can be

removed by Reidemeister move I, and if $\pm k, \pm k+1, \ldots \mp k, \mp k+1$ (or $\mp k+1, \mp k$), then crossings $k, k+1$ can be removed by Reidemeister move II.

All knot diagrams can be represented by Gauss codes, but in fact not all Gauss code sequences represent knot diagrams; for instance, the sequence $1, -2, -1, 2$ appears to be a consistent Gauss code of only two crossings, which cannot be simplified by Reidemeister moves, and no knot has $n = 2$. On attempting to draw a diagram with this code, one finds it would be necessary for there to be one extra crossing to allow the curve to return to its starting point. In fact, this is the Gauss code of the open diagram shown in Fig. 2 (e) of the main text, and Gauss codes for open diagrams, and their relation to virtual knots, is the subject of the next section.

It can be practically difficult to calculate the knot type of a diagram coming from a projection of a complicated 3D space curve, which may have many more crossings than its minimal number n. These crossings would represent local geometrical or biochemical features that do not affect the overall knot type; the knot diagrams found from closures of protein backbones often contain several hundred crossings. Our knot identification proceeds first by algorithmic simplification via removal of crossings, repeatedly applying Reidemeister moves I and II where they would remove crossings locally (Supplementary Fig. 1(a)), as discussed above. There is no known efficient method to produce minimal knot diagrams in this way as Reidemeister move III may also be essential to simplify the diagram but does not directly reduce the crossing number. In the case of protein backbones, this occasionally produces minimal diagrams but in most cases tens to hundreds of crossings remain.

The knot types of the simplified diagrams are calculated using *knot invariants*, quantities that depend only on the knot type but are calculated from the geometrical information of the curve, i.e. they can be calculated from only the information in a Gauss code and their value is invariant to Reidemeister moves. Much of mathematical knot theory is devoted to the study of knot invariants, and many types are known. For instance, the minimal crossing number discussed above is a knot invariant [4], but there is no simple algorithm to calculate it directly from a presentation of a knot. The minimal crossing number also demonstrates that most invariants do not perfectly distinguish knots [4], as multiple different knots can clearly have the same number of crossings in their minimal projections; for instance, both 5_1 and 5_2 in Fig. 2(a) have $n = 5$. More discriminatory invariants exist but are generally relatively difficult to calculate.

For knot identification we use knot invariants that can be calculated efficiently (ideally in low order polynomial time in the number of crossings), while still discriminating knots sufficiently well. In particular, we choose invariants which leave no ambiguity between the knots common on closure of proteins such as those in Fig. 2(a) of the main text. Some protein closures produce complex knots whose knot type cannot be uniquely identified using these efficient invariants, but these occur only rarely and do not impact our analysis. For classical knots, we employ only the *Alexander polynomial* $\Delta(t)$, which can be found as the determinant of a matrix whose rows and columns relate to the crossings of a projected diagram and can be easily constructed from a Gauss code [9]. Computing symbolic matrices numerically is relatively slow, and we instead use the values of $|\Delta(t)|$ evaluated at roots of unity $t = -1$, $t = \exp(2\pi i/3)$ and $t = i$, such that the calculation can be performed using floating point arithmetic (this does not introduce appreciable error). Each of these is individually a lesser knot invariant, but together they have discriminatory power comparable to the full Alexander polynomial up to at least 11 minimal crossings (certainly sufficient for the relatively simple knots that appear in protein chains).

Many knot invariants, including the Alexander polynomial, are available from standard online resources including the Knot Atlas [7] for all knots with up to 15 crossings, and KnotInfo [8] for a wider selection of invariants up to 12 crossings. Supplementary Table 1 shows values of $\Delta(t)$ at the roots of unity used above, for each of the simple knots that appear most commonly in protein chains.

Virtual Knots

Virtual knots are an extension to the theory of classical knots [10] which classify all topological objects formed of ordered crossings, which generalises the theory of knot diagrams while keeping a sense of isotopy through Reidemeister moves. In particular, this includes those orderings which cannot be realised as plane projections of (closed) space curves in $\mathbb{R}^3$. They can be thought of as the objects represented by the set of *all* Gauss codes, including sequences such as $1, -2, -1, 2$, which does not correspond to any closed knot diagram, as discussed above. In this sense, they provide a natural framework to describe *open* diagrams, with endpoints that cannot directly be joined, so do not correspond to classical knots but have knot-like structure in their sequence of ordered crossings.

Many concepts from classical knot theory naturally generalise to virtual knots, such as the distinction between prime and composite virtual knots (including composites with classical and virtual components). Virtual knots are

tabulated according to their minimum classical crossing number n [2], and they are denoted here as vn_m, following the tabulation of [2], as described in the main text. The simplest nontrivial virtual knot, $v2_1$, has $n = 2$, and Gauss code $1,-2,-1,2$. There are many more prime virtual knots for $n \geq 2$ than classical knots; complete tabulations only extend to virtual knots up to $n = 5$. There are also up to three distinct chiral symmetric partners of a given virtual knot (compared to at most one partner of opposite chirality for classical knots): a mirror reflection of the diagram preserving the classical crossing signs, an inversion where all classical crossing signs are flipped, and the combination of both mirrors. As with the classical knots, we identify all chiral partners of the same virtual knot type as equivalent.

[10] presents two further equivalent interpretations of virtual knots, both of which illustrate properties discussed in the main text. The first, convenient for diagrammatic representation, draws virtual knots as classical knot diagrams (without endpoints) but augmented with an additional crossing type at self intersection, the *virtual crossing*, denoted by a circle around the intersection (e.g. Fig. 2(b) of the main text). Virtual crossings do not have a sign and do not contribute to topological calculations, so the Gauss code follows only by considering the virtual diagram's classical crossings and ignoring virtual crossings entirely. In such virtual diagrams, virtual crossings can be manipulated by suitable generalisations of the classical Reidemeister moves, which can affect the configuration of virtual and classical crossings but do not change the virtual knot type; these moves are shown in Supplementary Fig. 1(b). In particular, virtual Reidemeister moves I and II can change the number of virtual crossings, and minimal virtual crossing number is an invariant of virtual knots; those with minimum virtual crossing number zero are the classical knots, which make up a subset of the generalised, virtual knots. We describe a knot here as virtual if the minimum number of virtual crossings is greater than zero.

The other interpretation of virtual knots is as closed knot diagrams drawn on surfaces with topology different to the standard plane of projection (equivalent to its one-point compactification, the 2-sphere), i.e. drawn on handlebodies with nonzero genus. Any virtual knot can be drawn as a knot diagram without virtual crossings on a surface of sufficiently high genus [10]. The virtual crossings previously described are then interpreted as a consequence of projection from the handlebody to a plane, in which case the virtual crossings are intersections of two strands from different bridges of the handlebody (likewise, a virtual knot diagram with virtual crossings can be made a knot diagram on a handlebody by replacing each virtual crossing with a handle which one strand passes 'along' and the other 'under' the handle). The minimum genus of any handlebody on which the virtual knot can be drawn defines the *virtual genus* (hereafter referred to as the genus, although this is distinctly different to the genus referred to in classical knot theory [4]) of the virtual knot, and is therefore 0 for classical knots while any virtual knot must have genus at least 1.

Here, we are considering 2D open diagrams as virtual knots, and these interpretations of virtual knots relate directly to virtual closure of open diagrams (formed by projection of open 3D chains) considered in the main text. The virtual closure of an open knot diagram corresponds to adding a closing arc between the open diagram's endpoints, where all intersections of this arc with the rest of the diagram make virtual crossings. All closure arcs are equivalent as they may be transformed to one another using virtual Reidemeister moves; the Gauss code only depends on the original open diagram, and does not change when the virtual crossings are altered. In fact, the virtual Reidemeister moves can be interpreted in terms of the endpoints of open diagrams, shown in Supplementary Fig. 1(c) in which the moves are effectively equivalent to different choices of closure.

It is possible to consider the open diagram in these terms alone (i.e. the open diagram is subject only to the three classical Reidemeister moves, but the endpoints are forbidden to pass over or under a strand creating (or removing) new crossings, Supplementary Fig. 1(d), otherwise the open diagram could be untangled to the trivial open curve); this would produce a *classical knotoid* [11], a topological object that encodes information about the topology of the open curve, but whose classes are not isomorphic to the virtual knots [1]. Representing knotoids by virtual knots loses some information – for instance it may not be clear, from a virtual diagram, which arc at a virtual crossing is the virtual closure arc (i.e. multiple, distinct knotoids give the same virtual knot). However, in our analysis, we opt to work with virtual knots since their tabulation, invariants and other properties are a lot better developed and understood than for knotoids, and therefore are more convenient for application without new mathematics. Only a small amount of information is apparently lost through the ambiguity of knotoids as virtual knots, which does not appear to unduly limit topological analysis; this can be considered as a similar simplification to ignoring the chirality of knots.

Since all the virtual crossings resulting from virtual closure necessarily occur sequentially along the same arc, the genus of virtual knots obtained by closing open diagrams is at most one. That is, all the virtual crossings of the diagram may be removed by adding a single handle to the surface on which it is drawn, in between the endpoints of the open

curve, and along which the closing arc runs. Not all genus one virtual knots can be represented in this way such that their virtual crossings occur sequentially; an example is shown in Supplementary Fig. 2(a), whose two virtual crossings can never be adjacent even under the application of (virtual) Reidemeister moves, although the knot can be drawn on a genus one surface sich as the planar diagram shown in Supplementary Fig. 2(b). The class of virtual knots that can be obtained from closures of open knot diagrams is therefore a subset of genus one virtual knots, whose minimal presentations pass around the torus exactly once in one generator direction, and at least once in the other. This is related to the homology of the curve as drawn on a genus one handlebody: for any such diagram we can associate an index with the number of times a curve wraps around the torus in each direction, and for a virtual knot these homology indices must be of the form $(\pm 1, j)$ for $|j| \geq 1$ (although this condition is not on its own sufficient due to the presence of more complex topologies with the same overall homology). We therefore refer to the virtual knots appearing as virtual closures of open curves as *minimally genus one virtual knots*.

The virtual knots of genus one were studied and tabulated by [3]. Their description involves a virtual knot invariant that is a generalisation of the Kauffman bracket polynomial with two variables a and x, calculated from the virtual knot diagram as drawn on the 2-torus. Each possible bracket smoothing of this diagram, s, is associated with a factor of $x^{\delta(s)}$, where $\delta(s)$ is the number of circles of nontrivial homology in a given smoothing. The polynomials for all minimally genus 1 virtual knots therefore have the form $xf(a)$, where $f(a)$ is a function of the knot which does not depend on x, and this property therefore allows all minimally genus one knots to be readily identified. The minimally genus one virtual knots of up to $n = 4$, in the genus one table [3] are, in the notation of that work: 2_1, 3_1, 4_1, 4_2, 4_3, 4_6, 4_7, 4_8 and 4_9. In the complete virtual knot table [2], the diagrams which are explicitly minimally genus one are: $v2_1$, $v3_2$, $v4_{12}$, $v4_{43}$, $v4_{65}$, $v4_{94}$ and $v4_{100}$. After comparing knot invariants between the two tabulations, we were unable to find a partner in [3] for the minimally genus one $v4_{12}$ (i.e. it appears to be an erroneous omission). Thus, from [3] we could identify three further minimally genus one virtual knots than the complete table, with this property also confirmed via the Kauffman bracket method; these correspond to $v4_{36}$, $v4_{37}$ and $v4_{64}$ in [2] (up to chiral mirrors). This relationship would be difficult to see by direct inspection of the diagram, and Supplementary Fig. 3 demonstrates the equivalence of the different presentations for $v4_{64}$, via a combination of virtual Reidemeister moves and planar isotopies. All other minimally genus one examples agree in the two tables, and we believe that this completes the full set of minimally genus one virtual knots with up to four classical crossings.

Just as with classical knots, we identify virtual knot types by calculating virtual knot invariants (which are, in many cases, generalisations of classical invariants, such as the Kauffman bracket polynomial already discussed). Typically it is more computationally expensive to discriminate virtual knots than classical knots of the same minimum crossing number n. The basic procedure of invariant calculation is similar to that of classical knots, although now virtual crossings may also be algorithmically removed via virtual Reidemeister moves I and II. This does not directly affect the classical crossings, but may allow more of them to be removed. The Alexander polynomial has a number of extensions in virtual knot theory; we work with the two variable *generalised Alexander polynomial* $\Delta_g(s,t)$ [12]. As with classical knots, the calculation is significantly faster evaluated at constant values of s and t, and we use the combinations ($s = -1$, $t = e^{2\pi i/3}$)), ($s = -1$, $t = i$) and ($s = e^{2\pi i/3}$, $t = i$). However, in contrast to classical knots, the generalised Alexander polynomial is not enough to distinguish the two simplest virtual knots possible from open curves, $v2_1$ and $v3_2$, as well as some other simple virtual knots (the next are $v4_{36}$ and $v4_{65}$, but although they are relatively simple these do not contribute significantly to any of our analysis). When necessary (but primarily in the case of $v2_1$ and $v3_2$), we resolve this ambiguity using the *Jones polynomial* $V(q)$ [13], which is a classical knot invariant that extends to virtual knots without modification. Since computation of the Jones polynomial takes exponential time in the number of crossings [4, 7], we compute it only at the constant $q = -1$ (sufficient to distinguish $v2_1$, $v3_2$, etc.), and only when our chosen values of $\Delta_g(s,t)$ are not sufficiently discriminatory to identify the virtual knot.

Virtual knot invariants for each of the virtual knots with up to four classical crossings can be found in the online knot table of [2] or, for the Kauffman bracket variant explained above, in [3]. Supplementary Table 1 further shows the values of Δ_g and V for each of the minimally genus one virtual knots in these tables, which together are clearly sufficient to distinguish all relevant knot types.

References

1. Gügümcü, N. & Kauffman, L. H. New invariants of knotoids. arXiv:1602.03579 (2016).

2. Green, J. & Bar-Natan, D. A table of virtual knots. URL https://www.math.toronto.edu/drorbn/Students/GreenJ/. Accessed Sep 2016, last updated Aug 2004.

3. Andreevna, A. A. & Matveev, S. V. Classification of genus 1 virtual knots having at most five classical crossings. *Journal of Knot Theory and Its Ramifications* **23**, 1450031 (2014).

4. Adams, C. C. *The Knot Book* (American Mathematical Society, 1994).

5. Rolfsen, D. (ed.) *Knots and Links* (AMS Chelsea Publishing, 1976).

6. Sossinsky, A. *Knots: mathematics with a twist* (Harvard University Press, 2002).

7. The Knot Atlas. URL http://katlas.org. Accessed Sep 2016.

8. Cha, J. C. & Livingston, C. Knotinfo: Table of knot invariants. URL http://www.indiana.edu/~knotinfo. Accessed Sep 2016.

9. Orlandini, E. & Whittington, S. G. Statistical topology of closed curves: Some applications in polymer physics. *Reviews of Modern Physics* **79**, 611–42 (2007).

10. Kauffman, L. H. Virtual knot theory. *European Journal of Combinatorics* **20**, 663–90 (1999).

11. Turaev, V. Knotoids. *Osaka Journal of Mathematics* **49**, 195–223 (2012).

12. Kauffman, L. H. & Radford, D. E. Bioriented quantum algebras and a generalized Alexander polynomial for virtual links. In *Diagrammatic Morphisms and Applications*, vol. 318 of *Contemporary Mathematics*, 113–40 (American Mathematical Society, 2003).

13. Jones, V. F. R. A polynomial invariant for knots and links via Von Neumann algebras. *Bulletin of the American Mathematical Society* **12**, 103–11 (1985).
